# Carbodiimide-Mediated Beckmann Rearrangement of Oxyma-B as a Side Reaction in Peptide Synthesis

**DOI:** 10.3390/molecules27134235

**Published:** 2022-06-30

**Authors:** Andrea Orlandin, Ivan Guryanov, Lucia Ferrazzano, Barbara Biondi, Francesca Biscaglia, Claudia Storti, Marzio Rancan, Fernando Formaggio, Antonio Ricci, Walter Cabri

**Affiliations:** 1 Fresenius Kabi iPSUM Srl, Via San Leonardo 23, 45010 Villadose, Italy; andrea.orlandin@fresenius-kabi.com (A.O.); walter.cabri@unibo.it (W.C.); 2 Institute of Chemistry, St. Petersburg State University, Peterhof, Universitetskij pr. 26, 198504 St. Petersburg, Russia; 3 Department of Chemistry “Giacomo Ciamician”, University of Bologna, Via Selmi 2, 40126 Bologna, Italy; lucia.ferrazzano4@unibo.it; 4 ICB, Padova Unit, CNR, Department of Chemistry, University of Padova, Via Marzolo 1, 35131 Padova, Italy; barbara.biondi@unipd.it (B.B.); francescabiscaglia@hotmail.it (F.B.); claudiastorti9@gmail.com (C.S.); fernando.formaggio@unipd.it (F.F.); 5 ICMATE, CNR, Department of Chemistry, University of Padova, Via Marzolo 1, 35131 Padova, Italy; marzio.rancan@unipd.it

**Keywords:** peptide, Beckman rearrangement, Oxyma-B, amino acid, coupling, racemization, glucagon, GLP-1

## Abstract

The suppression of side reactions is one of the most important objectives in peptide synthesis, where highly reactive compounds are involved. Recently, the violuric acid derivative Oxyma-B was introduced into peptide synthesis protocols as a promising additive to efficiently control the optical purity of the amino acids prone to racemization. However, we discovered a side reaction involving the Beckmann rearrangement of Oxyma-B during the coupling reaction, which compromises the yield and purity of the target peptides. Here, we present the investigation of the mechanism of this rearrangement and the optimization of the coupling reaction conditions to control it. These results can be taken into account for the design of novel efficient oxime-based coupling reagents.

## 1. Introduction

The rigid regulatory requirements imposed in recent years for the peptide active pharmaceutical ingredients promoted a continuous improvement of the methods for their manufacturing to obtain target compounds with high purity and yield [1,2,3]. To this aim, noticeable efforts have to be made in the development and optimization of the upstream and downstream processes. One of the most important steps of peptide synthesis is the peptide (amide) bond formation or coupling. Many efficient methods have been developed to carry out the coupling reaction with almost quantitative yield. Most of them are based on the activation of the carboxylic group of an amino acid into an electrophilic center to perform the following reaction with the amine nucleophile. This carboxylic group activation can be achieved by introducing an electron-withdrawing group at the carbonyl carbon atom, such as halide, azide, or more complex groups, which include an oxygen atom linked to a double-bonded carbon atom (O-C=), a cationic carbon (O-C^+^) or phosphorus (O-P^+^), or nitrogen adjacent to a double bond or double-bonded atom (O-N= and O-N-X=) [4]. However, carboxylic group activation can often facilitate a side-chain-induced racemization of sensitive amino acids, such as histidine, cysteine, serine, and threonine [5]. As a result, the manufactured peptides can contain diastereomeric impurities that are very difficult to separate without a significant decrease in yield. Various approaches have been proposed to suppress the loss of the optical purity of these susceptible amino acids during coupling reactions. For example, a careful selection of the side-chain protecting groups and a change in the solid support can noticeably decrease the amount of racemized product due to the electron-withdrawing effects and steric shielding [6,7,8,9,10,11,12].

**Figure 1:**
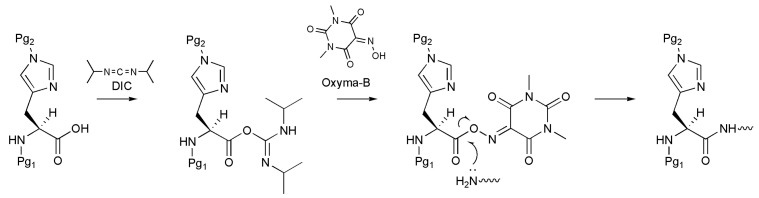
A scheme of Oxyma-B-mediated histidine coupling to the growing peptide chain (Pg_1_, Pg_2_ are protective groups).

One of the most efficient ways to prevent racemization is the use of an additive during the activation of the amino acid, such as 1-hydroxybenzotriazole (HOBt) or OxymaPure [13,14]. Despite the broad range of applicability, these additives show limited ability to suppress the racemization of susceptible amino acids [5]. In this regard, Jad et al. recently proposed the novel reagent Oxyma-B (1,3-dimethylvioluric acid) as an additive for the peptide coupling reaction (Figure 1) [15,16,17,18].

Oxyma-B can be easily prepared from 1,3-dimethylbarbituric acid by reaction with sodium nitrite in the presence of potassium hydroxide and acetic acid, and it affords more efficient control of the optical purity during the coupling reaction than other OxymaPure- and benzotriazole-based reagents. For example, Oxyma-B in combination with diisopropylcarbodiimide (DIC) was a more potent racemization suppressor than OxymaPure/DIC for His coupling in the synthesis of the H-Gly-His-Phe-NH_2_ tripeptide (1% vs. 3% of D-His isomer) [15].

Both OxymaPure and Oxyma-B belong to the class of oximes, which are known to be highly reactive compounds and can trigger various side reactions, such as Beckmann rearrangement to form substituted amides in the presence of strong acids and other activators of the oxime hydroxyl group [19,20,21]. To test the efficiency of Oxyma-B for industrial applications, we used this reagent for the preparation of various His-containing peptides. Surprisingly, in analyzing the high-performance liquid chromatography (HPLC) profiles of the isolated peptides, we found two impurities with the same molecular weight and overall content of up to 15%. The formation of these impurities can drastically decrease the yield of the target peptides, especially when multiple cycles of coupling with this additive have to be carried out. Accordingly, to understand the origin of this behavior not yet reported and to find the best reaction conditions to prevent the formation of these impurities, we synthesized them and studied their structures in detail.

## 2. Results and Discussion

**Figure 2:**
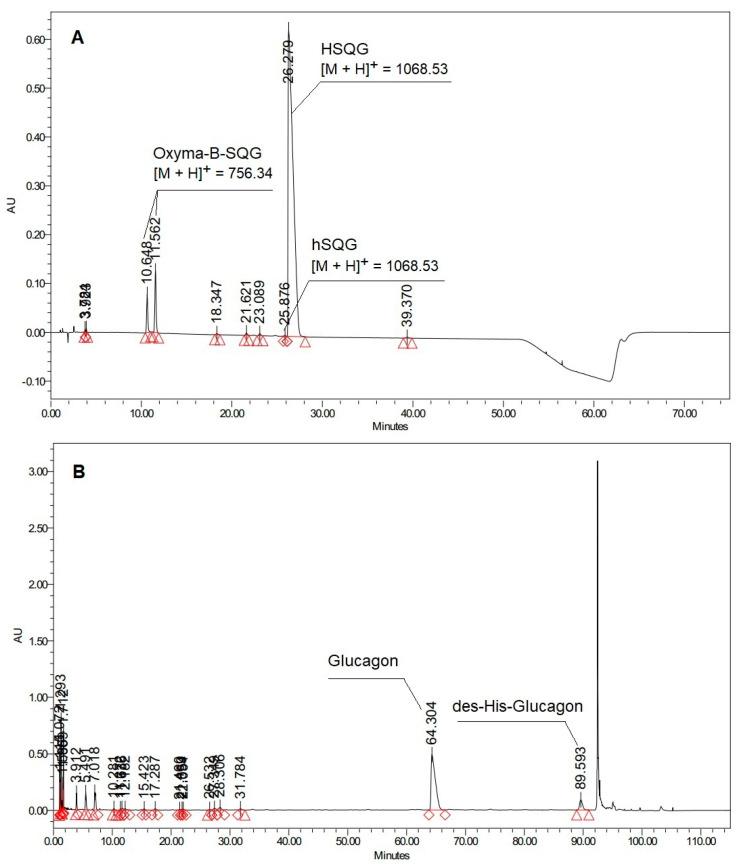
(**A**) HPLC profile of Boc-His(Trt)-Ser(*t*Bu)-Gln(Trt)-Gly-OH (hSQG refers to the D-His-HSQG diastereomer impurity of the protected HSQG sequence) (Analytical method 1, See the Experimental part); (**B**) HPLC profile of Glucagon prepared by the condensation of the HSQG tetrapeptide to the 5–29 fragment of Glucagon (Analytical method 2, See the Experimental part).

Histidine is present as the *N*-terminal amino acid of several commercially relevant peptides, such as Glucagon and GLP-1 analogs, which comprise the blockbuster drugs Liraglutide and Semaglutide currently used for diabetes treatment [22,23]. These peptides have a strong tendency to aggregate and to generate several side reactions during conventional step-by-step solid-phase synthesis, which noticeably complicates their preparation, particularly when the last residues have to be coupled [24]. To solve this problem, the use of a fragment condensation approach was selected as an excellent alternative, where protected peptide fragments are prepared separately and assembled to obtain the complete peptide sequence [25,26]. Accordingly, we carried out the synthesis of the *N*-terminal Glucagon fragment Boc-His(Trt)-Ser(*t*Bu)-Gln(Trt)-Gly-OH (abbreviated as HSQG later in the text), using DIC as a coupling reagent and Oxyma-B as an additive for the last coupling involving the His residue. As expected, Oxyma-B efficiently prevented the epimerization of histidine, resulting in only 0.09% of the D-His-diastereomer in the isolated HSQG sequence (Figure 2A). 

However, the HPLC profile of the isolated crude HSQG peptide showed the presence of two peaks of unexpected impurities with an overall area of 7.4% (with respect to the target product peak). The corresponding *m/z* value of these two impurities showed the same molecular weight, corresponding to the absence of the *N*-terminal histidine and the condensation of one molecule of Oxyma-B to the tripeptide H-Ser(*t*Bu)-Gln(Trt)-Gly-OH (with the concurrent elimination of one molecule of water). Furthermore, a gradual conversion of one HPLC peak to another one was observed over time, indicating that one of them could be an unstable intermediate. Lastly, the HPLC chromatogram of the crude Glucagon, which was prepared by on-resin condensation of fragment 5–29 with HSQG followed by the cleavage from the solid support, showed the presence of an equivalent amount (about 7.5%) of des-His-Glucagon, confirming the incomplete coupling of the His residue (Figure 2B).

**Figure 3:**
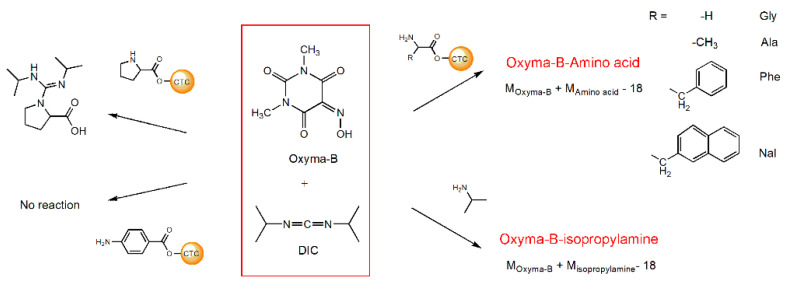
The reaction of Oxyma-B with various amino acids and isopropylamine in the presence of DIC.

To find an explanation for this unexpected result, we investigated the reaction using a series of model experiments with different amines and amino acids (Figure 3).

Several amino acids linked to 2-chlorotrityl chloride resin (CTC) were treated with the mixture of Oxyma-B and DIC in dimethylformamide. Oxyma-B-derivatives corresponding to the impurity observed in the synthesis of HSQG were found in the case of the α-amino acids with a primary amino group, as well as with isopropylamine (IPA) in solution (Figures S1 and S2). The 4-Aminobenzoic acid did not react with Oxyma-B, probably because of the low nucleophilicity of its amino function. Interestingly, in the case of amino acids with bulky side chains (phenylalanine and 3-(2-naphthyl)-alanine), two Oxyma-B derivatives with the same molecular weight were found, similarly to the tetrapeptide (Figure S1C,D). On the contrary, in the case of proline, only the corresponding guanidine derivative was observed, as it often occurs during peptide bond formation when there is a lack of the carboxylic component (data not shown) [27]. The study of the reaction of the mixture Oxyma-B/DIC with isopropylamine in dimethylformamide showed that the product of the reaction formed almost immediately and its amount increased with time with the consumption of the reagents (Figure S2).

**Figure 4:**
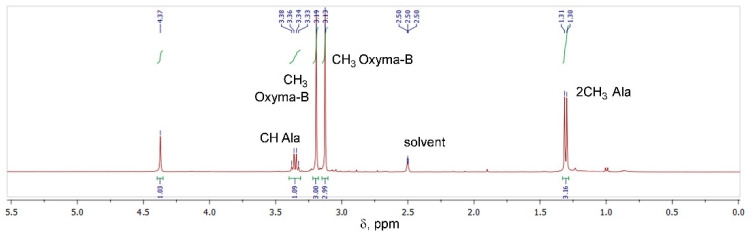
^1^H NMR of the compound formed in the reaction between Oxyma-B and alanine (400 MHz, DMSO-*d*_6_).

The Oxyma-B-derivatives of alanine and isopropylamine were purified by HPLC and their structures were analyzed by NMR spectroscopy (Figure S3). As expected, the characteristic peaks of alanine (α-Me, α-CH) and Oxyma-B (CH_3_-N) were present in the ^1^H NMR spectrum (Figure 4), confirming that the impurity contained both reagents in its structure.

**Figure 5:**
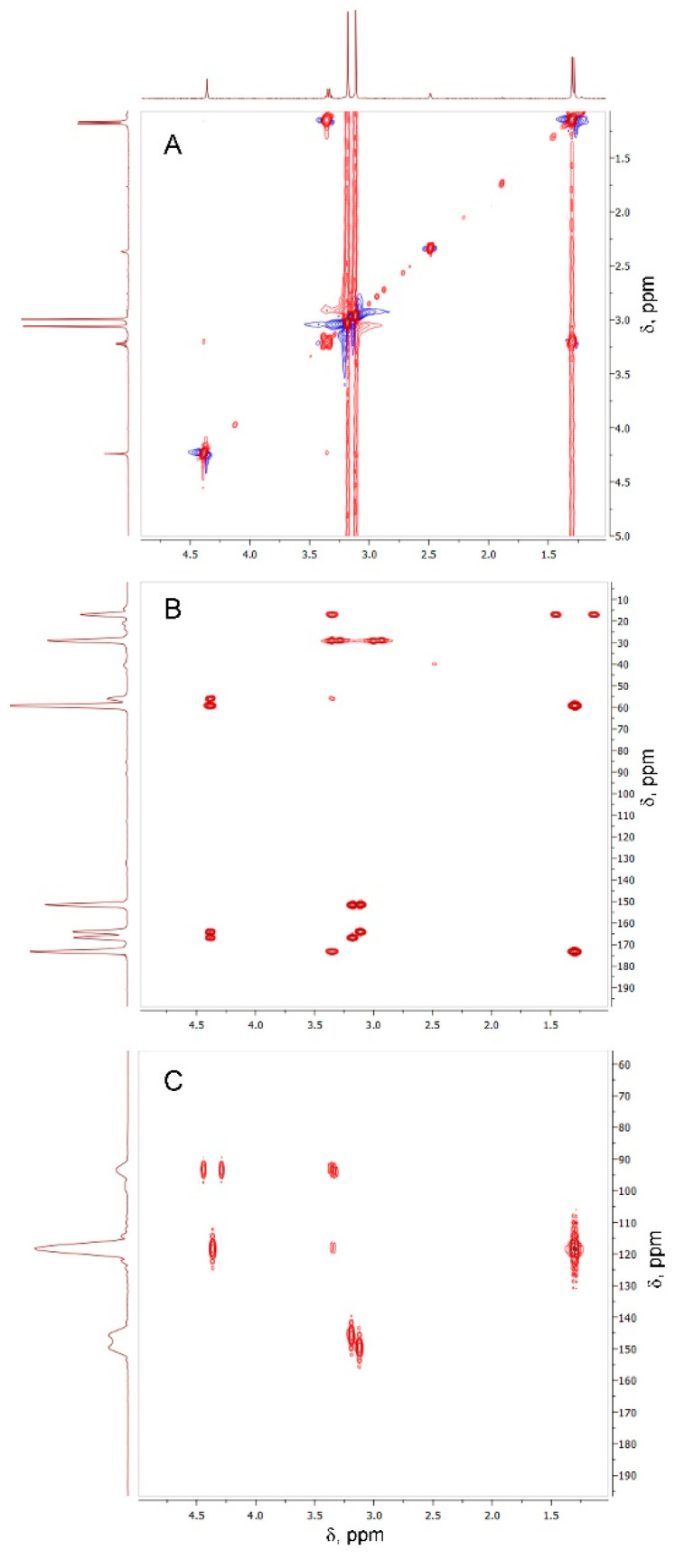
TOCSY (**A**), ^13^C-^1^H HMBC (**B**), and ^15^N-^1^H HMBC (**C**) spectra of the compound formed in the reaction between Oxyma-B and alanine (400 MHz, DMSO-*d*_6_).

However, the signal of the OH group of Oxyma-B at 14.72 ppm disappeared with the appearance of a new peak at 4.37 ppm, which suggested the involvement of the oxime group in the reaction (Figure S4). ^13^C-^1^H HSQC experiments showed the absence of its direct interaction with any carbon atom (Figure S5). Thus, the signal at 4.37 ppm could be attributed to the NH group in the proximity to the α-CH group of alanine (cross-peak C^α^H→NH at 3.34 ppm in the TOCSY spectrum, Figure 5A).

**Figure 6:**
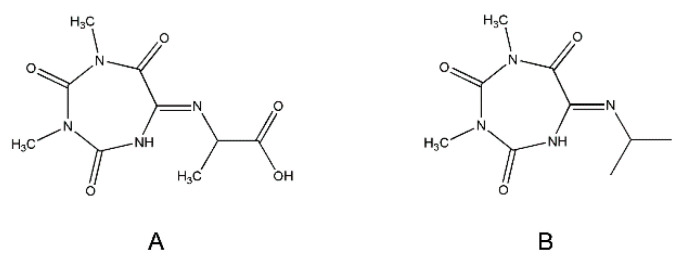
Structures of the Oxyma-B-derivatives of alanine (**A**) and isopropylamine (**B**).

Nevertheless, the *J*-coupling patterns of this proton and C^α^H in the ^1^H NMR spectrum (a singlet and a quartet, respectively) suggested their long-distance interaction. In the ^15^N-^1^H HMBC spectrum, four different types of nitrogen atoms were seen (Figure 5C). Two of them belonged to CH_3_-N of Oxyma-B (signals at 145.83 and 149.61 ppm), whereas the other two showed a close interaction with the C^α^H and the methyl group of alanine, as denoted by the intense cross-peaks at 93.43 and 118.65 ppm. Thus, the NMR analysis suggested the presence of the -NH-C=N-C^α^H(CH_3_)- fragment. In this case, the only structure that could fit the experimental data is a seven-membered ring formed from Oxyma-B (Figure 6A). Indeed, the ^13^C-^1^H HMBC spectrum confirmed this hypothesis (Figure 6B, Table S1).

The NMR analysis of the corresponding Oxyma-B-derivative of isopropylamine showed the same peak patterns and suggested the presence of a similar structure (Figure 6B, Figure S6–S9, Table S2). The presence of two isomers of the Oxyma-B derivatives of peptides and amino acids with bulky side chains, as well as their interconversion, can be explained by conformational preferences, such as imine–enamine tautomerism and a *cis-trans* transition.

**Figure 7:**
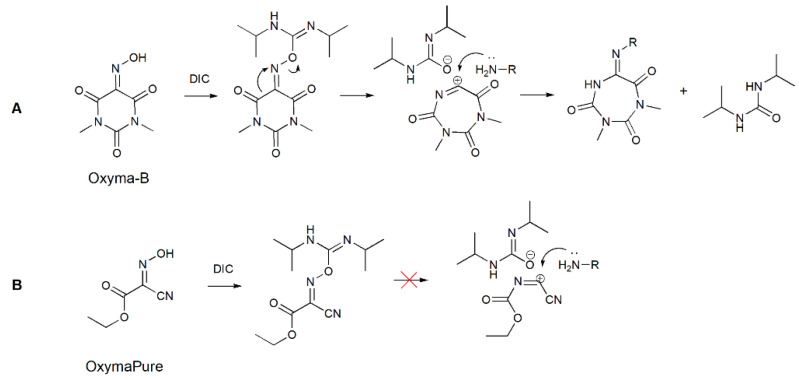
A possible mechanism of carbodiimide-mediated Beckmann rearrangement of Oxyma-B (**A**) and the reaction of OxymaPure with DIC (**B**).

The formation of such a cyclic structure could be a result of the Beckmann rearrangement of Oxyma-B in the presence of an amino group as a nucleophile. In this case, instead of the usually migrating alkyl groups, a carbonyl group in trans-position to the leaving OH formed a new C-N bond (Figure 7A) [20].

A similar reaction was used previously for the preparation of *N*-imidoylbenzotriazoles [28]. In that case, a sulfonate group was employed for the activation of the hydroxyl group of various oximes, which is necessary to trigger the rearrangement [29]. Many activators for the Beckmann rearrangement have been described, such as strong Lewis acids, BOP-Cl (bis(2-oxo-3-oxazolidinyl)phosphinic chloride), carbonyldiimidazole, organosulfonyl chlorides, and transition metal catalysts [30,31,32,33].

To confirm the structure of the Oxyma-B-derivatives formed in the reaction with amino acids, we prepared this compound in another way by treatment of alanine-loaded CTC resin with the tosylate ester of Oxyma-B. Indeed, the reaction led to the same product (Figure S10). Interestingly, no similar impurity was found in the case of OxymaPure [13]. Most probably, the presence of the cyano moiety (a strong electron-attracting group) in proximity to the oxime function prevented the rearrangement by the inhibition of the positively charged transition state (Figure 7). To understand the influence of the C=O group on the Beckmann rearrangement, we performed the reaction of isonitroso Meldrum’s acid (HONM) and DIC with alanine-loaded CTC resin [34]. In this case, no product with the desired molecular weight was observed but only a mixture of several impurities with higher mass values, which were difficult to identify, probably due to the reactivity of the ester function in the reaction media (Figure S11). On the contrary, cyclohexanone oxime did not react with isopropylamine or the alanine-loaded CTC resin, which confirmed the importance of the neighboring C=O group for the rearrangement.

**Figure 8:**
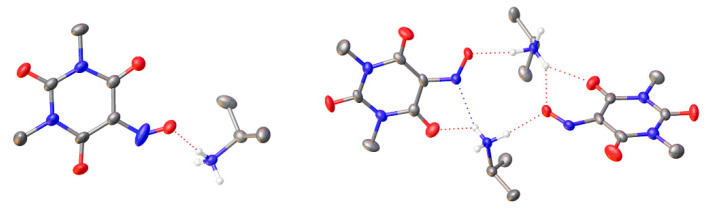
Crystal structure of Compound 1 (**left**) and Compound 2 (**right**). Color code: C gray, N blue, O red, H white. Anisotropic displacement ellipsoids for the non-H atoms are displayed at the 50% probability level. H-atoms of methyl and isopropyl groups as well as disordered parts were omitted for clarity. Hydrogen bonds are indicated by dotted red and blue lines.

To the best of our knowledge, no literature evidence of carbodiimide-mediated Beckmann rearrangement has been reported so far. From the experiments performed with structurally similar compounds, this kind of reaction is related to the peculiar structure of Oxyma-B. Indeed, the presence of multiple donor/acceptor sites leads to the inherent propensity of violuric acid derivatives to undergo chelation. Many brightly colored complexes with various metals and organoammonium ions have been separated and characterized [35]. To unravel the mechanism of the reaction, we evaluated if the formation of a complex of Oxyma-B with the *N*-terminal amino acids in the coupling mixture can favor the Beckmann rearrangement. Accordingly, we prepared the complex of Oxyma-B with isopropylamine; single crystals suitable for X-ray diffraction studies were obtained from both a hot water solution (Compound 1) and ethanol (Compound 2) (Figure 8, Figures S12 and S13).

Neither structure contained any solvent molecules of crystallization (water or ethanol). In the case of Compound 1, the asymmetric unit presented one isopropylammonium cation and one 1,3-dimethylviolurate anion, while the asymmetric unit of Compound 2 consisted of two isopropylammonium cations and two 1,3-dimethylviolurate anions. On the contrary to a previously reported crystal structure of diisopropylammonium violurate, no hydrogen bonds between 1,3-dimethylviolurate anions were observed because of the absence of N-H protons [36]. In our case, an extended array of hydrogen bonds was formed due to the interactions between the protonated nitrogen atom of the isopropylammonium cation and the N-O and C=O groups of the 1,3-dimethylviolurate anion. All of the hydrogen bond distances were similar and in the range of 1.88–2.14 Å (Tables S3 and S4).

The HPLC profile of the complex Oxyma-B with isopropylamine showed only a peak of Oxyma-B, probably because of its decomposition in the acidic eluents (Figure S14B). However, when the complex reacted with DIC, the formation of the same product as in the case of the reaction between the Oxyma-B/DIC mixture and IPA was observed (Figure S14A,C). The resulting chromatograms were very similar, confirming the possibility of the interaction of Oxyma-B with the *N*-terminal amino group, which may favor Beckmann rearrangement and the attachment of Oxyma-B to the growing peptide chain during amino acid coupling.

**Figure 9:**
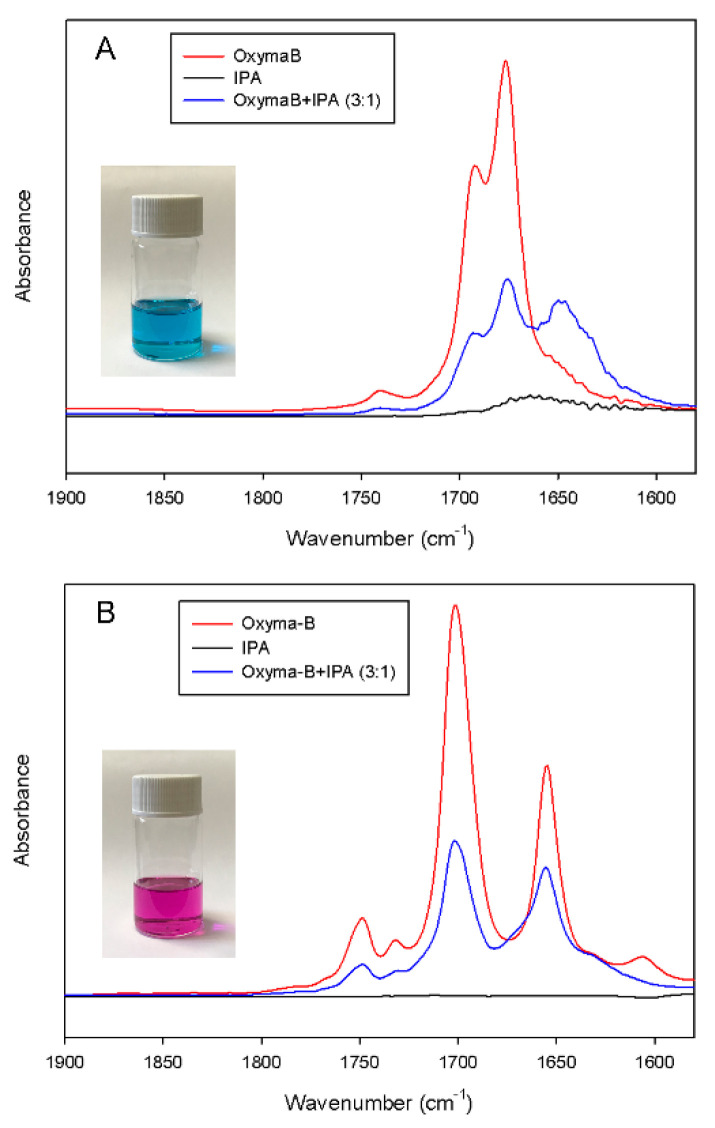
IR spectra of the mixture of Oxyma-B and isopropylamine (molar ratio 3:1) in acetonitrile (**A**) and in dichloromethane (**B**).

The formation of the complex between Oxyma-B and isopropylamine was also studied by IR spectroscopy. To avoid the superposition with the solvent IR bands, dimethyl sulfoxide was used as an aprotic polar solvent instead of dimethylformamide (Figure 9A).

**Figure 10:**
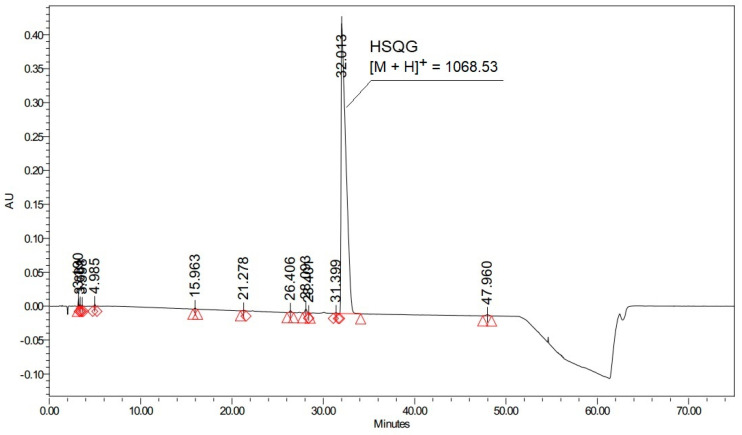
HPLC profile of Boc-His(Trt)-Ser(*t*Bu)-Gln(Trt)-Gly-OH. Oxyma-B/DIC mixture in dichloromethane was used for the activation of Boc-His(Trt)-OH (Analytical method 1, See the Experimental part).

The IR spectrum of free Oxyma-B showed two strong bands at 1692 cm^−1^ and 1676 cm^−1^, which can be attributed to the stretching vibrations of the carbonyl groups [37]. After the addition of isopropylamine in a 3:1 molar ratio of Oxyma-B/IPA (the ratio used for the reagents in the coupling mixture), a noticeable change in the position and intensity of the ν(C=O) band was observed. The shift to lower wavenumbers can indicate the presence of hydrogen bonding and complex formation. Interestingly, only a decrease in the intensity of the IR bands was seen in dichloromethane due to the dilution of the Oxyma-B solution with isopropylamine. It is likely that in the case of such a non-polar solvent, the complexation is less favorable, and this effect can lead to a different outcome of the coupling reaction. Moreover, the use of a non-polar solvent could also suppress the Beckmann rearrangement reaction [38]. Indeed, the substitution of dimethylformamide with dichloromethane as a solvent for the Oxyme-B-mediated coupling of histidine for the preparation of the HSQG tetrapeptide resulted in the absence of capping by Oxyma-B (Figure 10). In this case, almost no epimerization was observed in the peptide sequence as well.

Thus, the optimization of the reaction conditions for the preparation of activated esters with Oxyma-B noticeably improved the HPLC profiles and increased the yield of the target peptides.

## 3. Materials and Methods

### 3.1. Materials

Iris Biotech (Marktredwitz, Germany): *N*,*N*-dimetylformamide (DMF), dichloromethane (DCM), trifluoroacetic acid (TFA), 2-chlorotrityl chloride resin (CTC), 4-methylbenzhydryl bromide resin (Br-MBH), and Oxyma-B. Merck KGaA (Darmstadt, Germany): Acetonitrile (MeCN) for mass spectrometry (MS) (>99.9%), TFA for MS (>99.9%), diisopropyl ether, ammonium iodide, triisopropylsilane (TIS), isopropylamine (IPA), tosyl chloride, tetrabutylammonium hydroxide (TBA-OH), ethanedithiol (EDT), diisopropylethylamine (DIPEA), and hexafluoroisopropanol (HFIP). Carbosynth (San Diego, CA, USA): Ethyl (hydroxyimino) cyanoacetate (OxymaPure) and *N*,*N*′-diisopropylcarbodiimide (DIC). GL Biochem (Shanghai, China): Fmoc-Ala-OH (Fmoc, fluorenyl-9-methyloxycarbonyl), Fmoc-Ser(*t*Bu)-OH, Fmoc-Gln(Trt)-OH, Fmoc-Gly-OH, Boc-His(Trt)-OH, Fmoc-Phe-OH, Fmoc-Nal-OH, Fmoc-Asn(Trt)-OH, Fmoc-Met-OH, Fmoc-Leu-OH, Fmoc-Trp(Boc)-OH, Fmoc-Val-OH, Fmoc-Asp(tBu)-OH, Fmoc-Arg(Pbf)-OH, Fmoc-L-Asp(O*t*Bu)-L-Ser[psi(Me,Me)pro]-OH, Fmoc-Tyr(*t*Bu)-OH, Fmoc-Lys(Boc)-OH, and Fmoc-Thr(*t*Bu)-OH.

### 3.2. Methods

HPLC-MS chromatography: HPLC-MS analyses were performed on an Agilent Technologies 1200 instrument in tandem with or without Agilent 6530 mass accuracy Q-ToF.

Analytical method 1. Cortecs HPLC C18+ column (4.6 × 150 mm). Eluent A: TFA/H_2_O 0.05% *v*/*v*; Eluent B: MeCN; detection at 210 nm; gradient elution: 0 min-45% Eluent B; 5 min-45% Eluent B; 25 min-49% Eluent B; 50 min-51% Eluent B; 55 min-75% Eluent B; 60 min-85% Eluent B; 61 min-45% Eluent B; 75 min-45% Eluent B.

Analytical method 2. Ascentis Express ES-C18 column (4.6 × 150 mm). Eluent A: 5% MeCN in buffer A (19 mM TBA-OH and 5 mL phosphoric acid in 1 L of water); Eluent B: 60% MeCN in buffer B (7 mM TBA-OH and 1.5 mL phosphoric acid in 1 L of water); detection at 214 nm; gradient elution: 0 min-22% Eluent B; 1 min-22% Eluent B; 90 min-28% Eluent B; 91 min-100% Eluent B; 101 min-100% Eluent B; 102 min-22% Eluent B; 120 min-22% Eluent B.

Analytical method 3. C18 Pore-shell column (4.6 × 100 mm). Eluent A, TFA/H_2_O 0.1% *v*/*v*; eluent B: TFA/MeCN 0.1% *v*/*v*; detection at 220 nm; gradient elution: 0 min-1% Eluent B; 3 min-1% Eluent B; 33 min-30% Eluent B; 38 min-95% Eluent B; 40 min-1% Eluent B; 50 min-1% Eluent B.

Analytical method 4. C18 Pore-shell column (4.6 × 100 mm). Eluent A, TFA/H_2_O 0.1% *v*/*v*; Eluent B: TFA/MeCN 0.1% *v*/*v*; detection at 220 nm; gradient elution: 0 min-3% Eluent B; 3 min-3% Eluent B; 23 min-23% Eluent B; 25 min-5% Eluent B; 27 min-3% Eluent B; 37 min-3% Eluent B. 

Analytical method 5. C18 Pore-shell column (4.6 × 100 mm). Eluent A, TFA/H_2_O 0.1% *v*/*v*; Eluent B: TFA/MeCN 0.1% *v*/*v*; detection at 220 nm; gradient elution: 0 min-5% Eluent B; 3 min-5% Eluent B; 33 min-95% Eluent B; 38 min-95% Eluent B; 40 min-5% Eluent B; 50 min-5% Eluent B.

Preparative HPLC purification: The products were purified by preparative HPLC on an ÄKTA Pure purification system with UV detection at 224 nm using a C18 Jupiter column Phenomenex (21.2 × 250 mm) and Eluent A: 0.1% TFA solution in water; Eluent B: 0.1% TFA solution in MeCN. Elution was carried out using the following gradient: 0 min-1% Eluent B, 5 min-1% Eluent B; 65 min-20% Eluent B, 75 min-90% Eluent B, 85 min-90% Eluent B. Fractions with purity >97% were collected and lyophilized. The resulting compounds were analyzed by HPLC-MS.

NMR analysis: NMR spectra were recorded at 298 K in DMSO-d_6_ or DMF-d_7_ solution (Sigma Aldrich) on a Bruker Avance III HD spectrometer operating at 400 MHz, using the TOPSPIN 3.5 software package. All homonuclear spectra were acquired by collecting 512 experiments, which consisted of 64–80 scans and 2K data points. The spin systems were identified using CLEAN-TOCSY spectra with a 70-ms-long spin-lock pulse sequence [39,40]. For correct assignment of all resonances, heteronuclear ^1^H-^13^C and ^1^H-^15^N correlation spectra HSQC and HMBC were acquired [41,42].

FT-IR absorption measurements were performed on a Perkin Elmer model 1720 X FT-IR spectrophotometer, nitrogen flushed, equipped with a sample-shuttle device, at 2 cm^−1^ nominal resolution, averaging 100 scans.

X-ray crystallography: The data were collected using an Oxford Diffraction Gemini E diffractometer, equipped with a 2K × 2K EOS CCD area detector and sealed tube Enhance Cu X-ray source. The detector distance was set at 45 mm. The diffraction intensities were corrected for Lorentz/polarization effects as well as for absorption. Empirical multi-scan absorption corrections using equivalent reflections were performed with the scaling algorithm SCALE3 ABSPACK. Data reduction, finalization, and cell refinement were carried out through CrysAlisPro software. Accurate unit cell parameters were obtained by least-squares refinement of the angular settings of the strongest reflections, chosen from the whole experiment.

The structures were solved with Olex2 by using the ShelXT structure solution program by Intrinsic Phasing and refined with the ShelXL refinement package using least-squares minimization [43,44,45]. In the last cycles of refinement, non-hydrogen atoms were refined anisotropically. Hydrogen atoms were included in calculated positions, and a riding model was used for their refinement. In Compound 1, isopropylammonium and the O atom of the NO group were split into two parts, of which the occupancies were constrained to sum to 1.0. To better model these disordered groups, selected SADI, DFIX, and EADP were applied. In Compound 2, the two NO groups were split into two parts, of which the occupancies were constrained to sum to 1.0. To better model these disordered groups, SADI restraints were applied. Selected reflections with |Error/esd| >5 were omitted. Crystallographic table: Table S5. Deposition Numbers (2150915, 2150916) contain the supplementary crystallographic data for this paper. [46].

Crystal Data for Compound 1. C_9_H_16_N_4_O_4_, M_r_ = 244.26, triclinic, P-1 (No. 2), a = 4.8538(4) Å, b = 10.6747(7) Å, c = 12.3921(7) Å, *a* = 109.835(6)^°^, *b* = 90.063(6)^°^, *g* = 101.582(7)^°^, *V* = 590.01(7) Å^3^, *T* = 151(4) K, *Z* = 2, *Z’* = 1, *m*(Cu K*_a_*) = 0.925, 7574 reflections measured, 2131 unique (R_int_ = 0.0352), which were used in all calculations. The final *wR_2_* was 0.1880 (all data), and *R_1_* was 0.0584 (I ≥ 2 *s*(I)). CCDC number 2150915.

Crystal Data for Compound 2. C_18_H_32_N_8_O_8_, *M_r_* = 488.51, triclinic, *P*-1 (No. 2), a = 10.7726(5) Å, b = 11.1435(5) Å, c = 11.2572(3) Å, *a* = 85.873(3)^°^, *b* = 85.645(3)^°^, *g* = 65.501(4)^°^, *V* = 1224.93(9) Å^3^, *T* = 186(2) K, *Z* = 2, *Z’* = 1, *m*(Cu K*_a_*) = 0.891, 8159 reflections measured, 4411 unique (R_int_ = 0.0264), which were used in all calculations. The final *wR_2_* was 0.1519 (all data), and *R_1_* was 0.0549 (I ≥ 2 *s*(I)). CCDC number 2150916.

Synthesis of Boc-His(Trt)-Ser(tBu)-Gln(Trt)-Gly-OH: the synthesis of the peptide fragment was carried out by step-by-step SPPS using 2-chlorotrityl chloride resin (CTC resin) (117 g, medium loading 1.6 mmol/g). After swelling of the resin using 0.94 L of DCM, Fmoc-Gly-OH, and DIPEA (0.8 and 3 eq, with respect to the loading of the resin) in 0.47 L of DCM was added. The reaction mixture was stirred for 1 h, and the solvent was filtered off. The unreacted sites of the resin were capped using a solution of methanol (23 mL) and DIPEA (98 mL) in 0.58 L of DCM in 15 min. Then, the resin was treated with the solution of acetic anhydride (53 mL) and DIPEA (98 mL) in 0.55 L of DMF for 15 min and washed with DMF (3 × 0.7 L). The loading of the resin was checked by UV adsorption measurement of the solution after Fmoc deprotection and was found to be 1.08 mmol/g. Then, Fmoc-l-Gln(Trt)-OH and Fmoc-l-Ser(*t*Bu)-OH (2-fold excess with respect to the loading of the resin) were pre-activated by DIC (2 eq) and Oxyma Pure (2 eq) in 5 min at 10 °C and coupled consecutively to the resin in 90 min. In the case of Boc-l-His(Trt)-OH, DIC (2 eq) and Oxyma-B (2 eq) in DCM or DMF were used. The intermediate Fmoc deprotection was carried out using a 20% solution of piperidine in DMF (2 × 0.6 L, 10 min each) with the subsequent washing of the resin with DMF (4 × 0.6 L). After the completion of the synthesis, the resin was washed with DCM and dried. The protected peptide was cleaved from the resin by treatment with 0.7 L of 1.5% TFA in DCM for 2 min, and the solution was filtered into 0.13 L of 10% solution of pyridine in methanol. The procedure was repeated 4 times. The combined solutions were concentrated to 30% of the volume. The concentrated solution was extracted 3 times with 0.4 L of water, and the aqueous part was discharged. The protected tetrapeptide was precipitated by adding 8 L of MTBE, filtered, washed three times by MTBE, and dried.

His coupling in DMF: Yield: 81 g (60.1%); HPLC purity: 91.5%;

His coupling in DCM: Yield: 98 g (72.9%); HPLC purity: 98.0%;

C_63_H_69_N_7_O_9_: [M + H]^+^_calc_ = 1068.52, [M + H]^+^_exp_ = 1068.53.

Synthesis of Glucagon: the synthesis was carried out by a solid-phase approach on MBH-Br resin (1.6 mmol/g, 10 g). After swelling of the resin in 200 mL of DCM, Fmoc-Thr(*t*Bu)-OH (0.6 mmol/g of resin), KI (1.5 equiv), and DIPEA (1.5 equiv) were added, and the suspension was stirred for 20 h. The resin was washed with DCM (3 × 60 mL), the residual free hydroxyl groups were capped with 60 mL of 0.5 M acetic anhydride solution in DMF in 15 min, and the resin was washed with DMF (3 × 60 mL). The Fmoc group was removed by treatment with a 20% piperidine solution in DMF (2 × 60 mL, 10 min each) and washed with DMF (4 × 60 mL, 2 × 5 min, and 2 × 10 min). The substitution degree was checked by UV adsorption measurement of the solution collected after Fmoc deprotection and was found to be 0.6 mmol/g. Fmoc-protected amino acids (fourfold excess with respect to the loading of the resin) were pre-activated before coupling with DIC (4 equiv.) and OxymaPure (4 equiv.) for 3 min at 10 °C and consecutively coupled to the resin for 90 min. Fmoc-l-Asp(O*t*Bu)-L-Ser[psi(Me,Me)pro]-OH (3 equiv. respect to the loading of the resin) and Boc-His(Trt)-Ser(*t*Bu)-Gln(Trt)-Gly-OH (2 equiv.) were coupled at 40 °C in 180 min after pre-activation with DIC and OxymaPure (3 eq. and 2 eq., respectively) for 15 min. After each coupling, the unreacted amino groups were capped using a 0.5 M solution of acetic anhydride in DMF. The intermediate Fmoc group removal was carried out with a 20% piperidine solution in DMF (2 × 60 mL, 10 min for each cycle) and washed with DMF (4 × 60 mL, 2 × 5 min, and 2 × 10 min). At the end of the synthesis, the resin was washed with DMF (3 × 60 mL) and DCM (3 × 60 mL) and then dried. The dry peptidyl resin was suspended in 200 mL of the TFA/TIS/H_2_O/EDT/Methionine/NH_4_I mixture (92.5:2:2:2:1:0.5 *v*/*v*/*v*/*v*/*w*/*w*) pre-cooled to 0–5 °C and stirred for 4 h at room temperature. The resin was filtered off, and old diisopropyl ether (800 mL) was added to the solution. The obtained pale-yellow suspension was stirred at 0–5 °C, and the solid was filtered, washed 3 × in 200 mL of diisopropyl ether, and dried in vacuo. Yield: 5.2 g (27%), HPLC purity: 57.9%.

Synthesis of Oxyma-B-derivatives of amino acids: Fmoc-protected amino acids (1.60 mmol) and DIPEA (0.28 mL, 1.60 mmol) were dissolved in 5 mL of DCM and added to CTC resin (0.5 g, medium loading 1.6 mmol/g) swelled in DCM. The reaction mixture was stirred for 2 h, and the resin was filtered and washed with DCM (3 × 5 mL). The unreacted sites of the resin were capped using 5 mL of the DCM/DIPEA/methanol mixture (*v*/*v*/*v* 17/2/1) and then with a 10% solution of acetic anhydride in DCM. Fmoc deprotection was carried out using a 20% solution of piperidine in DMF (2 × 5 mL, 10 min each) with the subsequent washing of the resin with DMF (4 × 10 mL). Then, the resin was treated with a solution of Oxyma-B (0.44 g, 2.40 mmol) and diisopropylcarbodiimide (0.38 mL, 2.40 mmol) overnight and washed with DMF (3 × 10 mL) and DCM (4 × 10 mL) and then dried. The product was cleaved from the resin by a 30% solution of HFIP in DCM (2 × 5 mL, 30 min each), and the organic solvent was evaporated to yield an oily residue.

Glycine: [M + H]^+^ = 234.0; HPLC purity: 92.5%; yield: 0.14 g (74%);

Alanine: [M + H]^+^ = 257.0; HPLC purity: 91.1%; yield: 0.16 g (78%);

Phenylalanine: [M + H]^+^ = 333.1; HPLC purity (two isomers): 65.1%; yield: 0.12 g (74%);

3-(2-Naphtyl)-alanine: [M + H]^+^ = 383.1; HPLC purity (two isomers): 73.7%; yield: 0.14 g (46%);

Proline: [M + H]^+^ = 242.0; traces.

Synthesis of the Oxyma-B-derivative of isopropylamine: Oxyma-B (100 mg, 0.54 mmol) was dissolved in 2 mL of DMF, and DIC (85 μL, 0.54 mmol) and isopropylamine (44 μL, 0.54 mmol) were added to the solution. The reaction mixture was stirred overnight (the formation of the product was followed by HPLC) and lyophilized. The product was purified by preparative HPLC. 

Oxyma-B-IPA: [M + H]^+^ = 227.1; HPLC purity: 98.5%; yield: 52 mg (43%).

Synthesis of the tosylate ester of Oxyma-B: Et_3_N (37.70 mL, 0.2705 mol) was added dropwise to a stirred solution of Oxyma-B (10.00 g, 0.0541 mol) and TsCl (12.37 g, 0.0649 mol) in 50 mL of DCM at 0 °C. The reaction mixture was stirred for 1 h and diluted with water (150 mL), and the product was extracted with DCM (3 × 50 mL). The combined organic layers were washed with water (3 × 100 mL) and brine (2 × 100 mL) and then dried. The solvent was evaporated, and the product was used without further purification. 

[M + H]^+^_calc_ = 340.1; [M + H]^+^_exp_ = 340.0;

HPLC purity: 95%; yield: 15.6 g (85%).

Synthesis of the Oxyma-B-derivative of alanine via the tosylate ester of Oxyma-B: Alanine-loaded CTC-resin, which was prepared as described previously, was treated with the solution of tosyl ester of Oxyma-B (814 mg, 2.40 mmol) in 5 mL of DMF overnight, washed with DMF (3 × 10 mL) and DCM (4 × 10 mL) and then dried. The product was cleaved from the resin by a 30% solution of HFIP in DCM (2 × 5 mL, 30 min each), and the organic solvent was evaporated to yield an oily residue.

[M + H]^+^ = 257.0; HPLC purity: 42.6%; yield: 0.11 g (54%).

Reaction between isonitroso Meldrum’s acid and alanine-loaded CTC: Isonitroso Meldrum’s acid was prepared as described in [34].

Alanine-loaded CTC-resin (0.5 g), which was prepared as described previously, was treated with the solution of isonitroso Meldrum’s acid (0.42 g, 2.40 mmol) and diisopropylcarbodiimide (0.38 mL, 2.40 mmol) overnight and washed with DMF (3 × 10 mL) and DCM (4 × 10 mL) and then dried. The product was cleaved from the resin by a 30% solution of HFIP in DCM (2 × 5 mL, 30 min each), and the organic solvent was evaporated to yield an oily residue.

## 4. Conclusions

Despite a huge number of approaches proposed so far, the development of more efficient strategies for the synthesis of peptides with amino acids prone to racemization is still an open topic. Recently, a very promising derivative of violuric acid, Oxyma-B, was introduced into peptide synthesis protocols due to its ability to afford amide bond formation with a very high yield and to suppress the loss of chirality. However, despite its superior efficiency, we discovered a side reaction that involves Oxyma-B and leads to the capping of the growing peptide chain, drastically lowering the yield of the desired product. The spectroscopic analysis of the compounds formed in this reaction allowed us to determine their structure and to suggest a possible mechanism of their formation through a carbodiimide-mediated Beckmann rearrangement, which is probably favored by the peculiar structure of Oxyma-B. Changes in the coupling reaction conditions allowed us to suppress this side reaction almost completely and to obtain peptides with excellent purity and high yield. Thus, the results described herein can noticeably improve the existing methods of peptide synthesis and can be considered in the development of novel oxime-based reagents.

## Data Availability

Not applicable.

## Supplementary Materials

The following supporting information can be downloaded at: https://www.mdpi.com/article/10.3390/molecules27134235/s1. HPLC profiles of the intermediates, final compounds and reaction mixtures, NMR spectra, a scheme of the preparation of the Oxyme-B derivative of alanine via tosyl chloride, crystal structures, and hydrogen bond information for Compounds 1 and 2.

## Sample Availability

Samples of the compounds are not available from the authors.
