# Supplementary material for: Carbodiimide-Mediated Beckmann Rearrangement of Oxyma-B as a Side Reaction in Peptide Synthesis
Source: Molecules. 2022 Jun 30;27(13):4235. doi: 10.3390/molecules27134235 (PMC9268172; doi:10.3390/molecules27134235)
Supplement: Supplementary file 1 [file molecules-27-04235-s001.zip › molecules-1794799-supplementary.pdf]

Supplementary information

Carbodiimide-mediated Beckmann rearrangement of Oxyma-B as a side reaction in peptide synthesis

Andrea Orlandin[1], Ivan Guryanov[1,2,*], Lucia Ferrazzano[3], Barbara Biondi[4], Francesca Biscaglia[4], Claudia Storti[4], Marzio Rancan[5], Fernando Formaggio[4], Antonio Ricci[1,*], Walter Cabri[1,3]

[1] Fresenius Kabi iPSUM Srl, via San Leonardo 23, Villadose (RO), 45010 Italy
[2] *Institute of Chemistry, St. Petersburg State University, St. Petersburg, Peterhof, Universitetskij pr. 26, 198504 Russia*
[3] Department of Chemistry "Giacomo Ciamician", Alma Mater Studiorum, University of Bologna, via Selmi, 2-40126, Bologna, Italy
[4] ICB, Padova Unit, CNR, Department of Chemistry, University of Padova, Padova, via Marzolo 1, 35131 Italy
[5] ICMATE, Padova Unit, CNR, Department of Chemistry, University of Padova, Padova, via Marzolo 1, 35131 Italy

Table of Contents

**Figure S1**	HPLC profiles of the products of the reaction between Oxyma-B and amino acids ……………..…	S2
**Figure S2**	The study of the reaction between the mixture of Oxyma-B/DIC and isopropylamine in DMF........	S3
**Figure S3**	HPLC profiles of the Oxyma-B-derivatives of Ala and IPA after preparative purification………..	S4
**Figure S4**	^{1}H NMR of Oxyma-B.………………………………………………………………………	S4
**Figure S5**	^{13}C-^{1}H HSQC spectrum of Oxyma-B-derivative of alanine ……………………………………...	S5
**Table S1**	NMR chemical shifts of Oxyma-B-derivative of alanine ……………………..…………………	S6
**Figure S6**	^{1}H NMR spectrum of Oxyma-B-derivative of isopropylamine.……………………………………..	S7
**Figure S7**	^{13}C NMR of Oxyma-B-derivative of isopropylamine.…………………………………..……...	S7
**Figure S8**	TOCSY spectrum of Oxyma-B-derivative of isopropylamine ………….………………………….	S8
**Figure S9**	^{13}C-^{1}H HMBC spectrum of Oxyma-B-derivative of isopropylamine…….……………………….	S8
**Table S2**	NMR chemical shifts of Oxyma-B-derivative of isopropylamine…………………………………..	S9
**Figure S10**	A scheme of the preparation of the Oxyme-B derivative of alanine *via* tosyl chloride and HPLC profiles of the products prepared by tosylate method and DIC-mediated method…………………..	S10
**Figure S11**	HPLC profiles of the products prepared from isonitroso Meldrum's acid and alanine-loaded CTC resin………………………………………………………………………………………………..	S10
**Figure S12**	Crystal structure of Compound 1………………………………………………………………	S11
**Table S3**	Hydrogen Bond information for Compound 1……………………………………………………	S11
**Figure S13**	Crystal structure of Compound 2………………………………………………………………	S12
**Table S4**	Hydrogen Bond information for Compound 2……………………………………………………	S12
**Table S5**	Crystal data and structure refinement……………………………………………………………….	S13
**Figure S14**	HPLC profiles of the reaction mixture Oxyma-B/DIC and IPA, complex Oxyma-B/IPA and reaction mixture of the complex Oxyma-B/IPA and DIC…………………………….……………...	S14

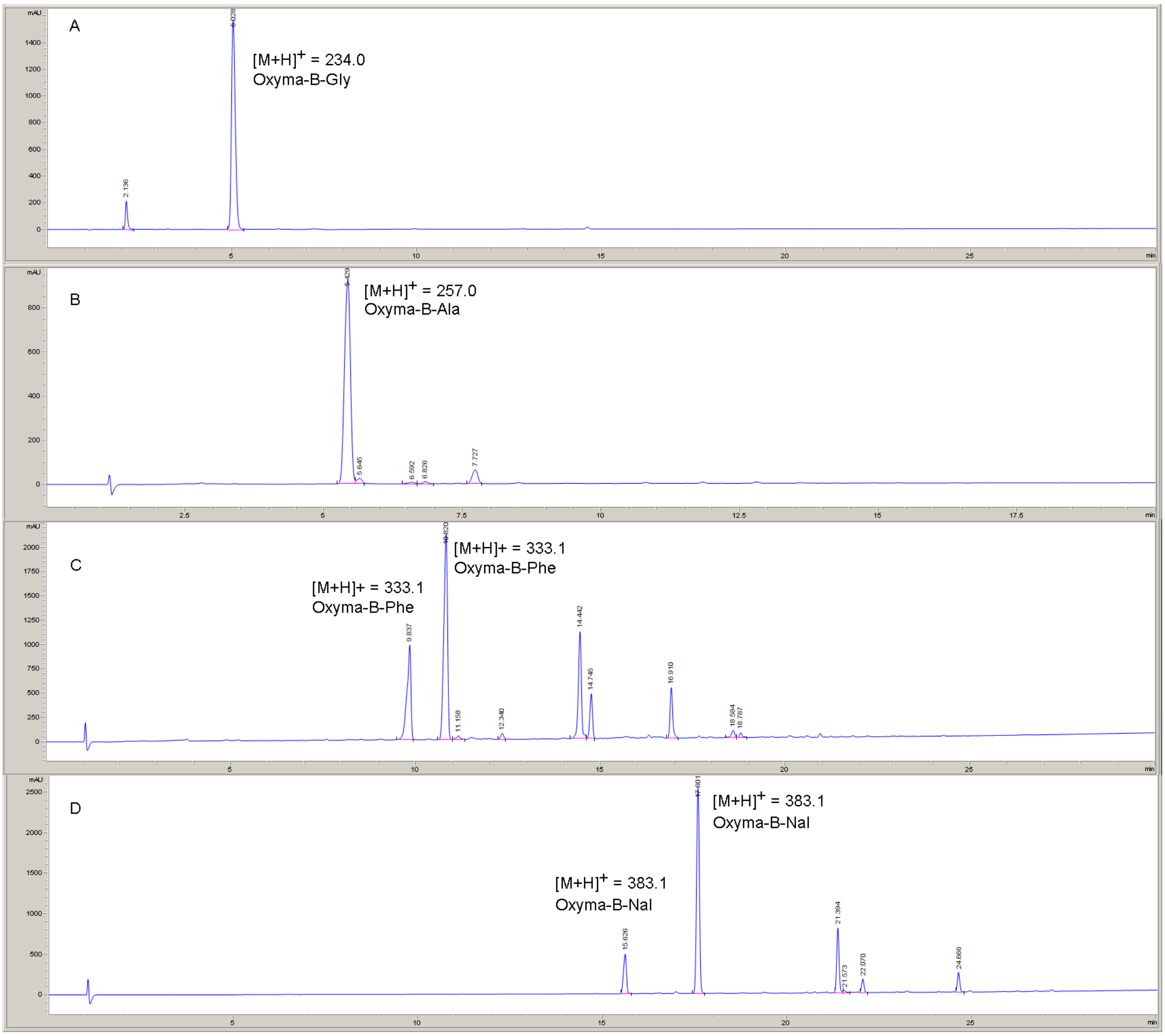

Figure S1. HPLC profiles of the products of the reaction between Oxyma-B and glycine (A), alanine (B), phenylalanine (C), and 3-(2-naphthyl)-alanine (D) (Analytical method 3 for A, 4 for B, 5 for C and D, see Experimental part).

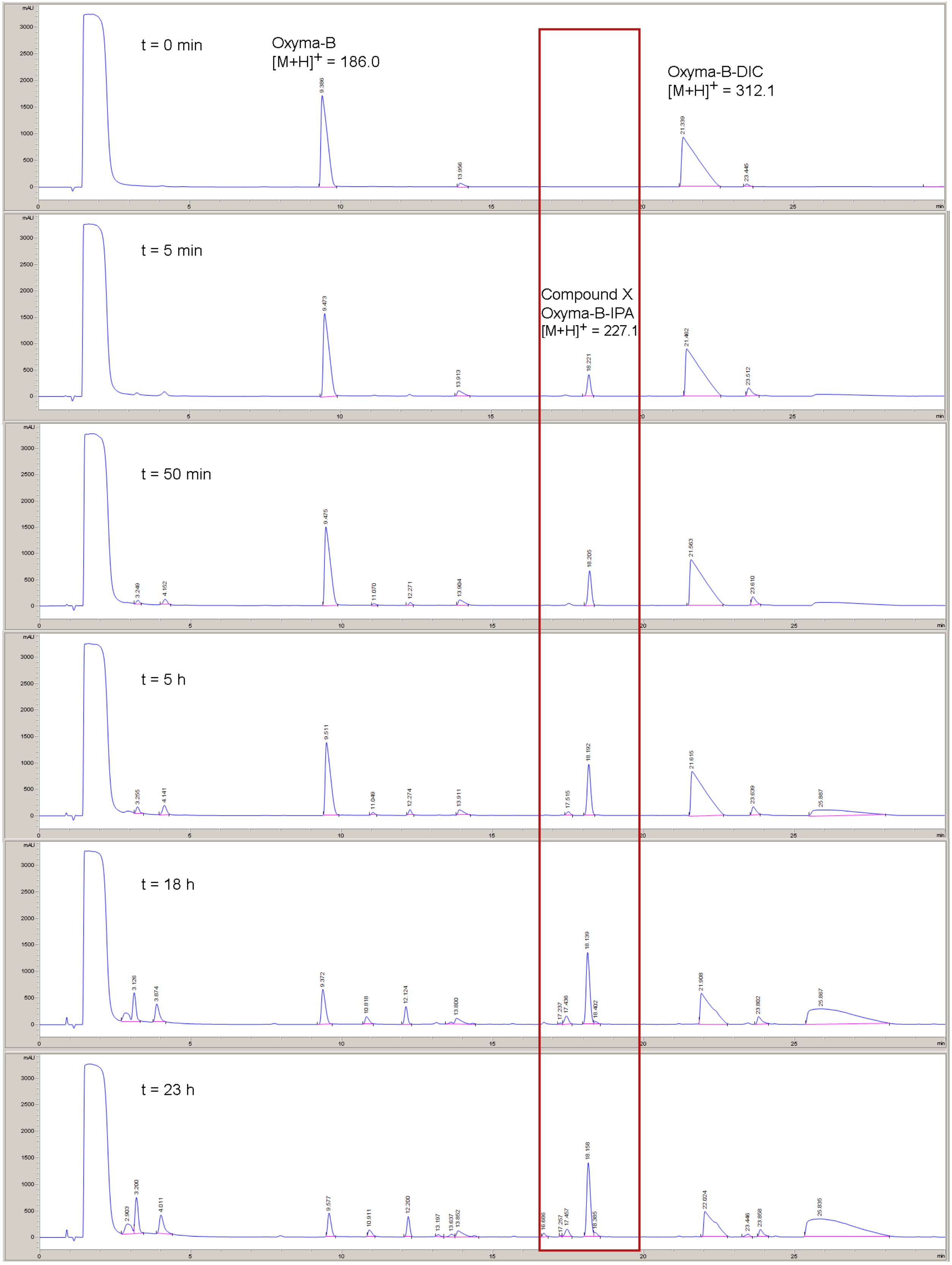

Figure S2. The study of the reaction between the mixture of Oxyma-B/DIC and isopropylamine in dimethylformamide (Analytical method 3, see Experimental part).

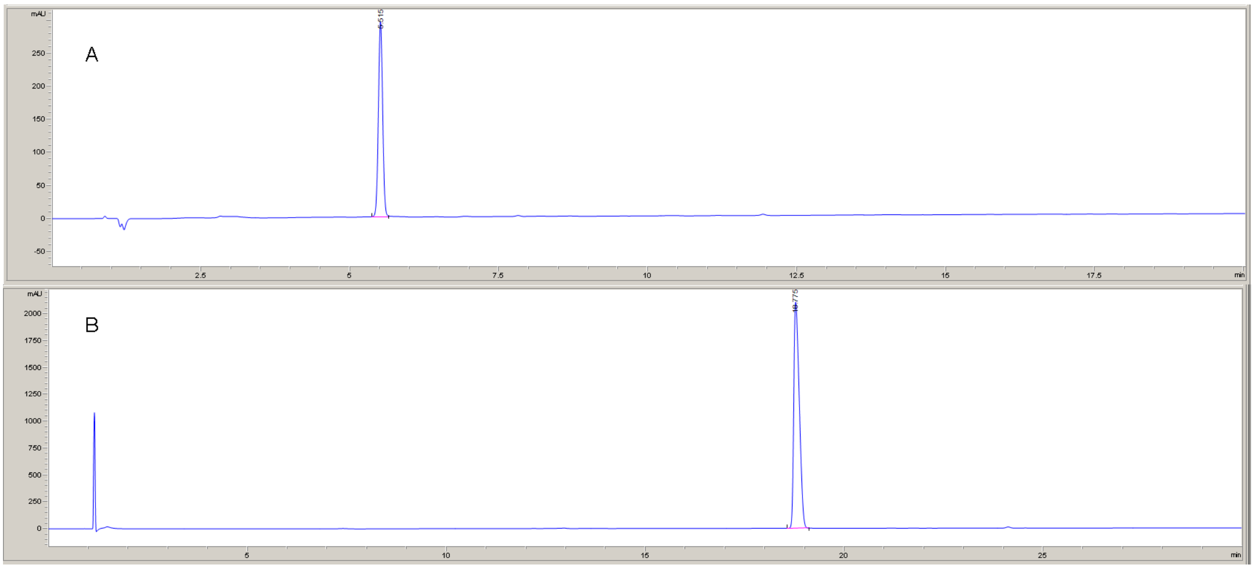

Figure S3. HPLC profiles of the Oxyma-B-derivatives of alanine (A), and isopropylamine (B) after preparative purification (Analytical method 3 for A, 4 for B, see Experimental part).

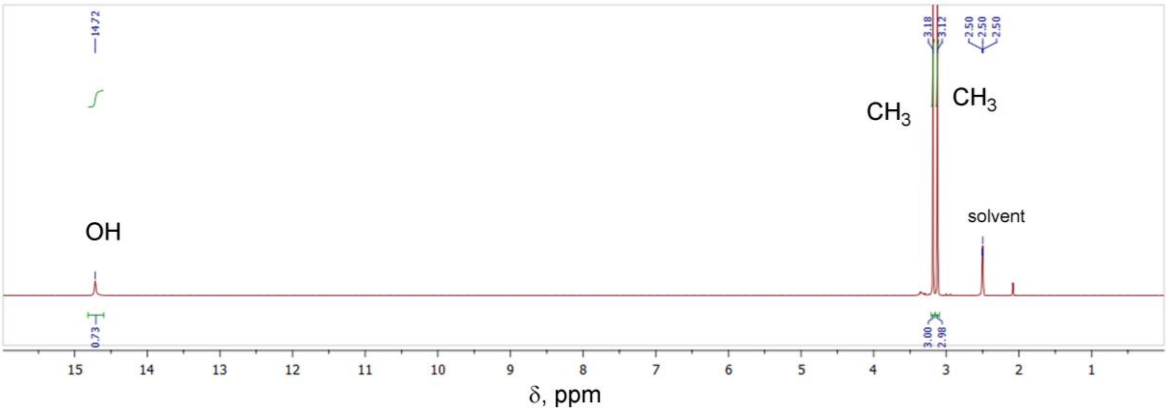

Figure S4. ^{1}H NMR of Oxyma-B (200 MHz, DMSO-d_6).

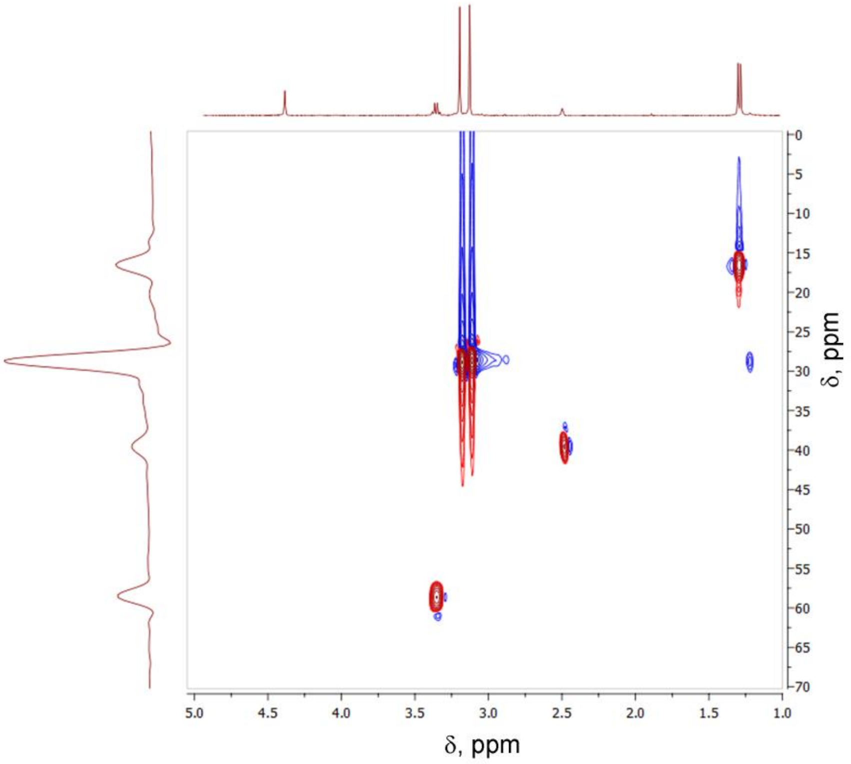

Figure S5. ^{13}C-^{1}H HSQC spectrum of Oxyma-B-derivative of alanine (400 MHz, DMSO-d_6).

Table S1. NMR chemical shifts of Oxyma-B-derivative of alanine.

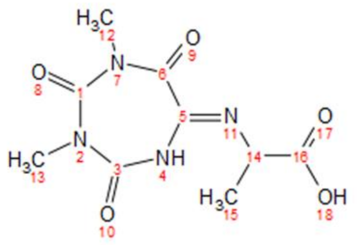

Atom	Compound A, δ (ppm)
H12, H13	3.13 (s); 3.19 (s)
H4	4.37 (s)
H15	1.30 (d, *J* = 4 Hz)
H14	3.34 (q, *J* = 8 Hz)
C1	151.59
C3, C6	164.14; 166.85
C5	56.06
C12, C13	29.11
C14	58.55
C15	17.08
C16	173.24
N2, N7	145.83; 149.61
N11	118.65
N4	93.43

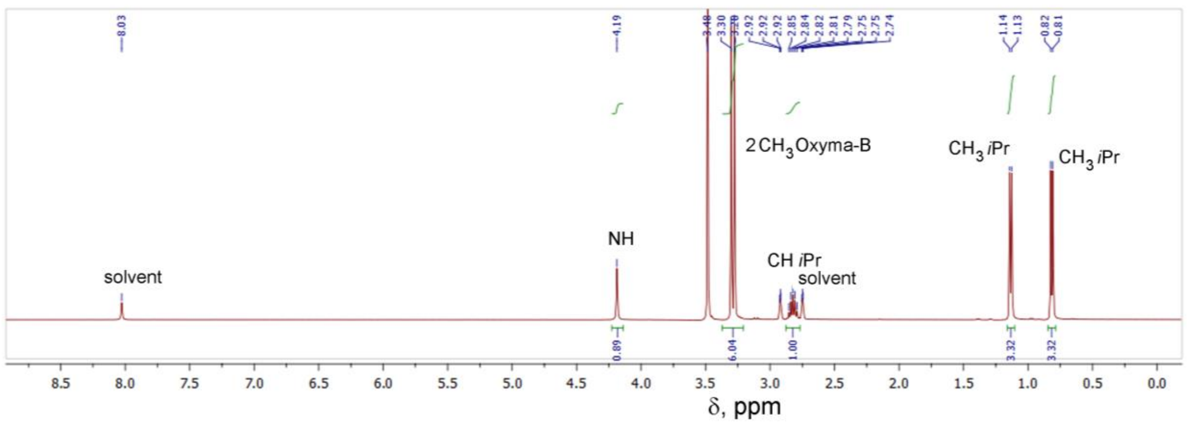

Figure S6. ^{1}H NMR spectrum of Oxyma-B-derivative of isopropylamine (400 MHz, DMF-d_7).

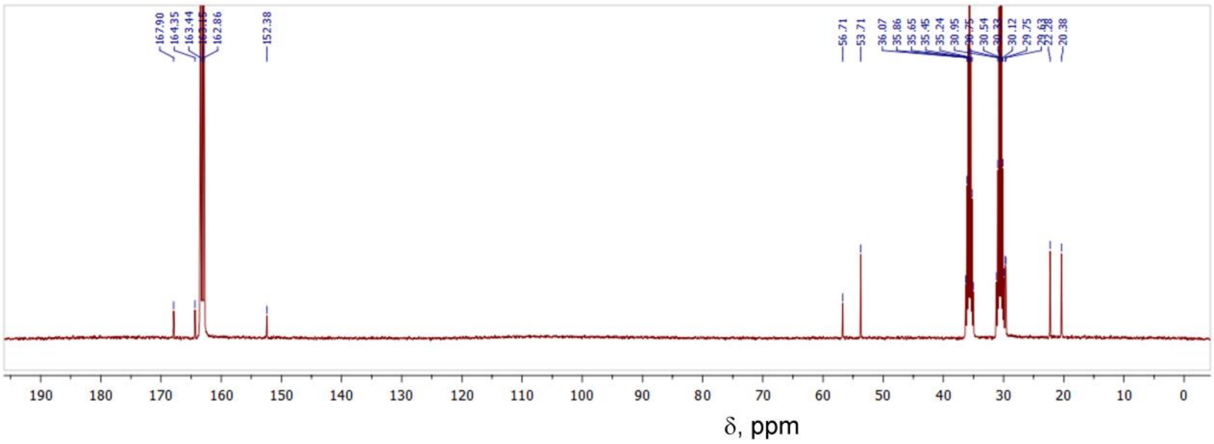

Figure S7. ^{13}C NMR spectrum of Oxyma-B-derivative of isopropylamine (400 MHz, DMF-d_7).

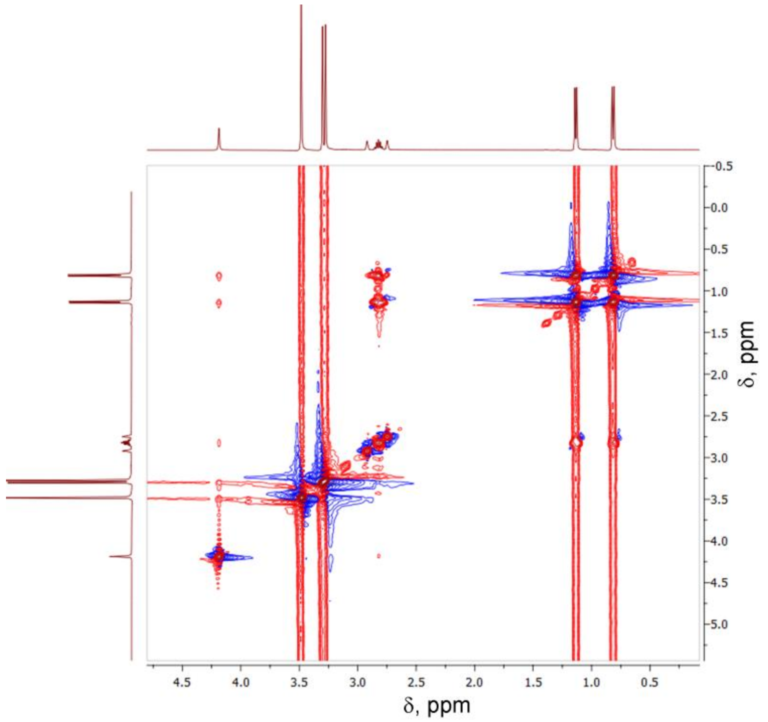

Figure S8. TOCSY spectrum of Oxyma-B-derivative of isopropylamine (400 MHz, DMF-*d*$_7$).

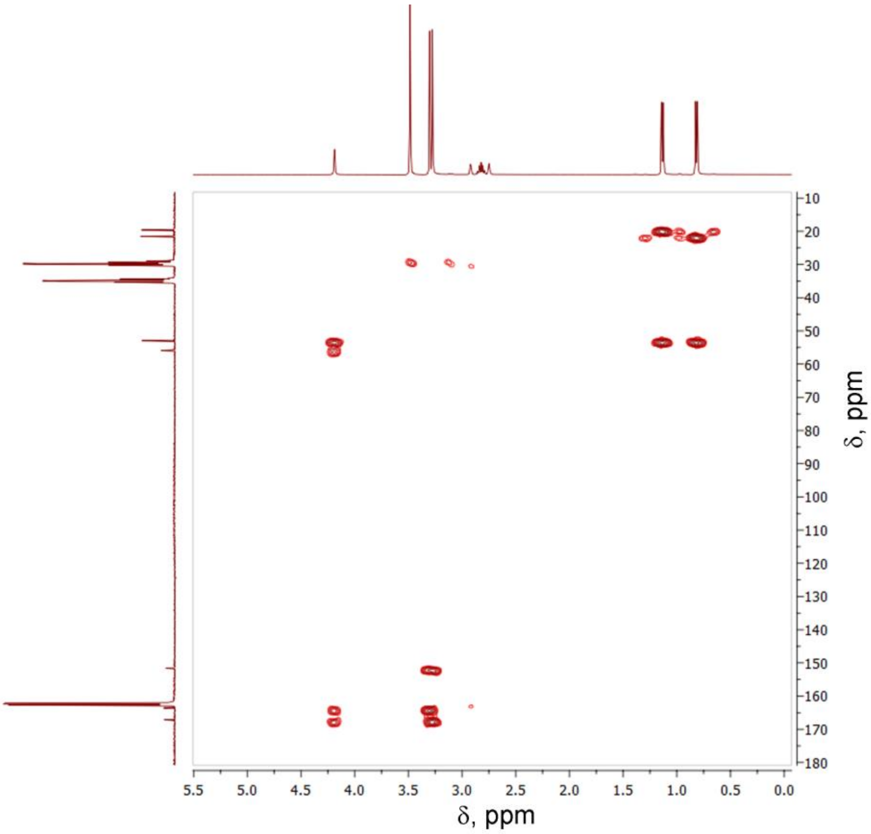

Figure S9. ^{13}C-^{1}H HMBC spectrum of Oxyma-B-derivative of isopropylamine (400 MHz, DMF-*d*$_7$).

Table S2. NMR chemical shifts of Oxyma-B-derivative of isopropylamine.

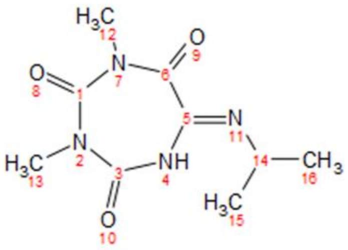

Atom	δ (ppm)
H12, H13	3.28 (s); 3.30 (s)
H14	2.82 (sept, *J* = 8 Hz)
H4	4.19
H15, H16	1.13; 0.81 (d, *J* = 4 Hz)
C1	152.38
C3, C6	167.90; 164.35
C5	56.71
C12, C13	29.54
C14	53.71
C15, C16	22.28; 20.38

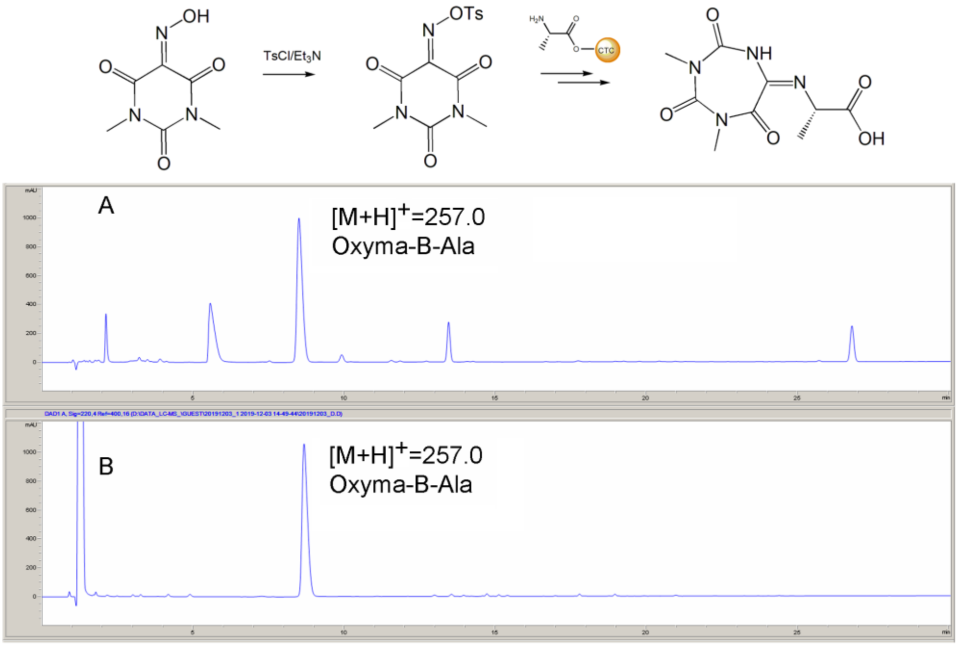

Figure S10. A scheme of the preparation of the Oxyme-B derivative of alanine *via* tosyl chloride and HPLC profiles of the products prepared by tosylate method (A) and DIC-mediated method (B) (Analytical method 5, see Experimental part).

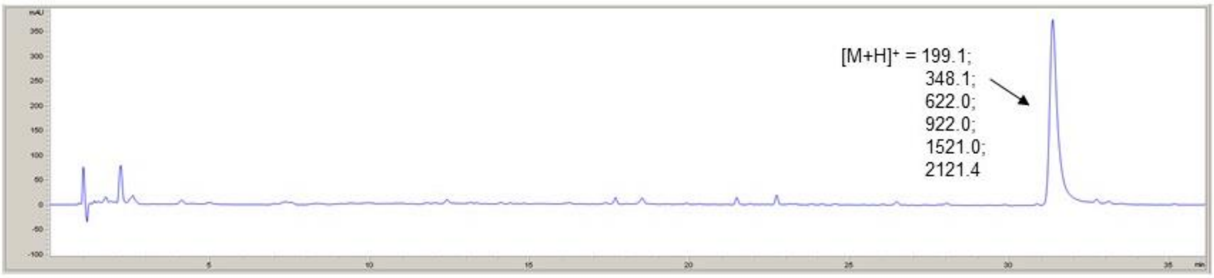

Figure S11. HPLC profile of the product of the reaction between isonitroso Meldrum's acid and alanine-loaded CTC resin (Analytical method 3, see Experimental part).

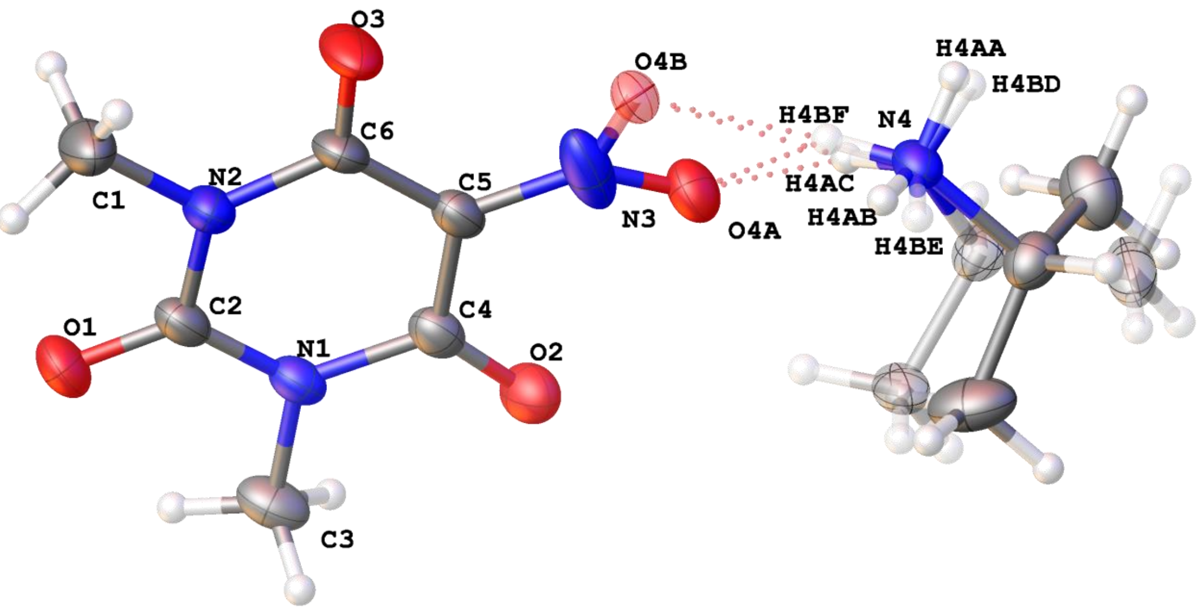

Figure S12. Crystal structure of Compound 1. Color code: C gray, N blue, O red, H white, disordered parts are translucent. Anisotropic displacement ellipsoids for the non-H atoms are displayed at the 50% probability level. H-atoms of methyl and isopropyl groups are omitted for clarity. Hydrogen bonds are indicated by dotted red and blue lines.

Table S3. Hydrogen Bond information for Compound 1.

D	H	A	d(D-H)/Å	d(H-A)/Å	d(D-A)/Å	D-H-A/deg
N4	H4BD	O3[1]	0.91	1.98	2.867(2)	165.3
N4	H4BF	O4B	0.91	1.91	2.819(4)	177.0
N4	H4AB	O4A[2]	0.91	2.08	2.815(3)	137.1
N4	H4AC	O4A	0.91	1.81	2.700(3)	164.7

[1]-x,-y,1-z; [2]-1+x,+y,+z

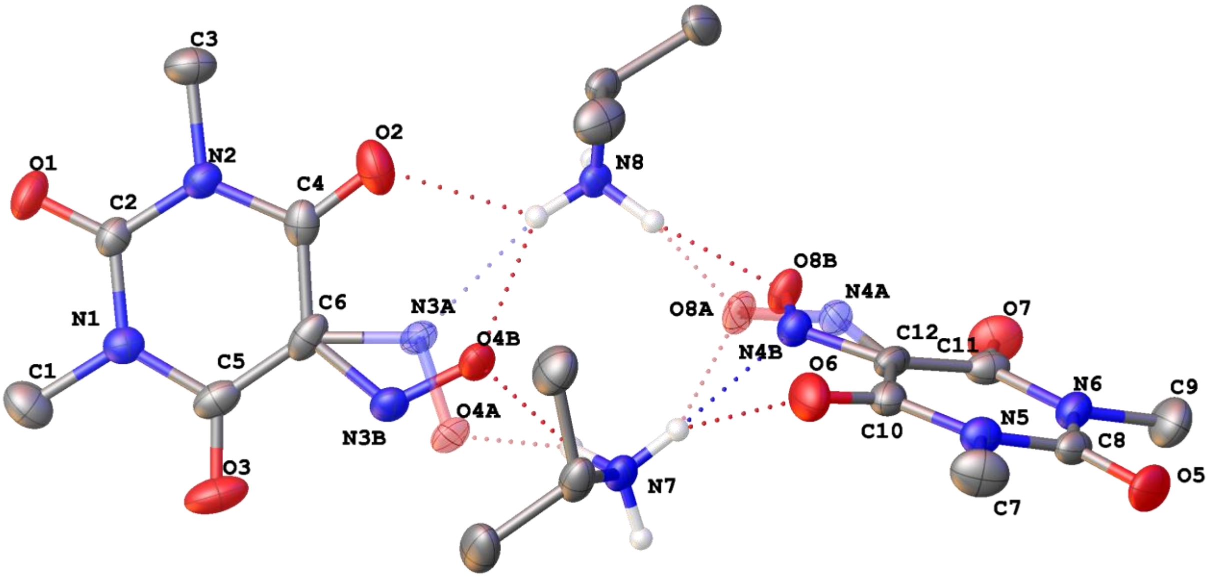

Figure S13. Crystal structure of Compound 2. Color code: C gray, N blue, O red, H white, disordered parts are translucent. Anisotropic displacement ellipsoids for the non-H atoms are displayed at the 50% probability level. H-atoms of methyl and isopropyl groups are omitted for clarity. Hydrogen bonds are indicated by dotted red and blue lines.

Table S4. Hydrogen Bond information for Compound 2.

D	H	A	d(D-H)/Å	d(H-A)/Å	d(D-A)/Å	D-H-A/deg
N7	H7D	O4B[1]	0.91	1.96	2.747(8)	143.3
N7	H7E	O8B	0.91	2.14	2.865(8)	136.0
N7	H7F	O4A	0.91	1.92	2.816(4)	166.0
N7	H7F	O4B	0.91	1.92	2.804(8)	163.1
N8	H8A	O4A	0.91	2.09	2.887(3)	145.4
N8	H8B	O8A	0.91	1.92	2.818(3)	171.1
N8	H8B	O8B	0.91	1.88	2.726(9)	154.4
N8	H8C	O7[2]	0.91	2.13	2.849(3)	135.1
N8	H8C	O8A[2]	0.91	2.06	2.816(3)	140.3

[1]1-x,1-y,1-z; [2]1-x,1-y,2-z

Table S5. Crystal data and structure refinement.

**Compound**	**1**	**2**
Formula	$C_9H_{16}N_4O_4$	$C_9H_{16}N_4O_4$
$D_{calc.}$/ g cm^{-3}	1.375	1.375
m/mm^{-1}	0.925	0.925
Formula Weight	244.26	244.26
Colour	dark pink	dark pink
Shape	irregular-shaped	irregular-shaped
Size/mm^3	0.18×0.12×0.08	0.18×0.12×0.08
T/K	151(4)	151(4)
Crystal System	triclinic	triclinic
Space Group	*P*-1	*P*-1
a/Å	4.8538(4)	4.8538(4)
b/Å	10.6747(7)	10.6747(7)
c/Å	12.3921(7)	12.3921(7)
a/°	109.835(6)	109.835(6)
b/°	90.063(6)	90.063(6)
g/°	101.582(7)	101.582(7)
V/Å^3	590.01(7)	590.01(7)
Z	2	2
Z'	1	1
Wavelength/Å	1.54184	1.54184
Radiation type	Cu K_a	Cu K_a
Q_{min}/°	3.803	3.803
Q_{max}/°	68.729	68.729
Measured Refl's.	7574	7574
Indep't Refl's	2131	2131
Refl's I≥2 *s*(I)	1680	1680
R_{int}	0.0352	0.0352
Parameters	193	193
Restraints	20	20
Largest Peak	0.345	0.345
Deepest Hole	-0.268	-0.268
GooF	1.070	1.070
wR_2 (all data)	0.1880	0.1880
wR_2	0.1706	0.1706
R_1 (all data)	0.0717	0.0717
R_1	0.0584	0.0584
CCDC	2150915	2150916

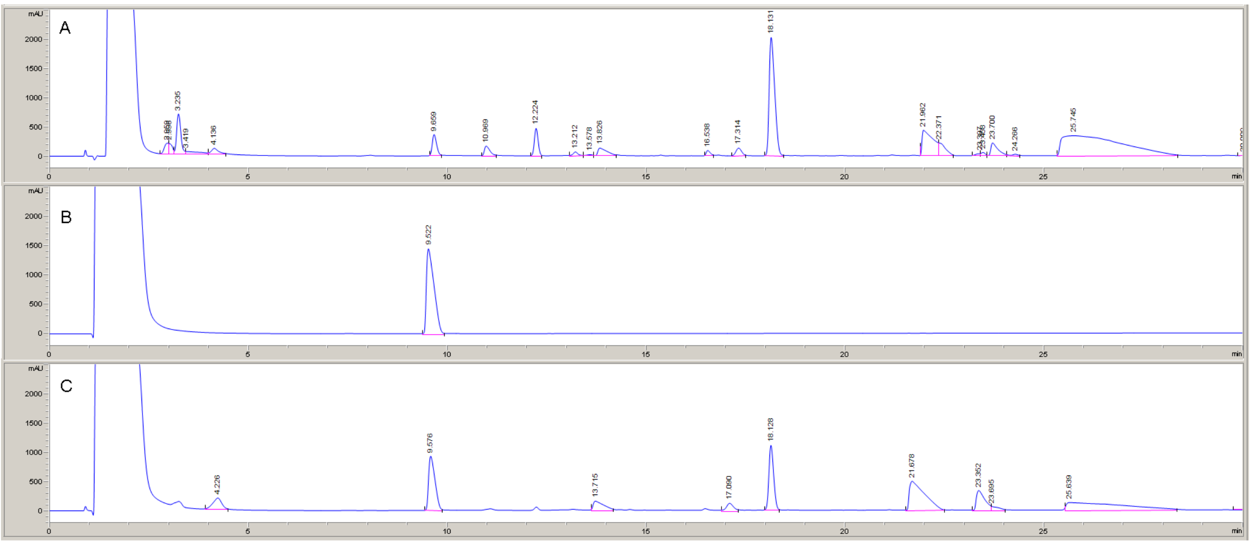

Figure S14. HPLC profiles of the reaction mixture Oxyma-B/DIC and IPA (3:1) in DMF after 24 h (A); complex Oxyma-B/IPA (B) and reaction mixture of the complex Oxyma-B/IPA and DIC (1:1) in DMF after 24 h (C) (Analytical method 3, see Experimental part).
